# Polysaccharide from *Trichosanthes kirilowii* Maxim ameliorates diphenoxylate-induced functional constipation in mice

**DOI:** 10.3389/fmicb.2025.1672600

**Published:** 2025-09-23

**Authors:** Min Jiang, Chuangchuang Wang, Jian Chen, Guozhen Wu, Wei Liu, Tao Li, Xiao Wang

**Affiliations:** ^1^School of Pharmaceutical Sciences, Shandong University of Traditional Chinese Medicine, Jinan, China; ^2^Shandong Engineering Research Center for Innovation and Application of General Technology for Separation of Natural Products, Shandong Analysis and Test Center, Qilu University of Technology (Shandong Academy of Sciences), Jinan, China; ^3^Department of Traditional Chinese Medicine, Central Hospital Affiliated to Shandong First Medical University, Jinan, China; ^4^Key Laboratory for Natural Active Pharmaceutical Constituents Research in Universities of Shandong Province, School of Pharmaceutical Sciences, Qilu University of Technology (Shandong Academy of Sciences), Jinan, China

**Keywords:** functional constipation, polysaccharide, *Trichosanthes kirilowii* Maxim, gut microbiota, neurotransmitters

## Abstract

**Introduction:**

*Trichosanthes kirilowii* Maxim has been well-documented for its pharmacological effects in alleviating constipation. Notably, polysaccharides, a class of natural macromolecules, demonstrate significant therapeutic potential for constipation management. Nevertheless, the preventive effect of *Trichosanthes kirilowii* polysaccharide (TKP) against functional constipation and its underlying mechanisms remain poorly understood.

**Methods:**

In this study, a mouse model of diphenoxylate-induced functional constipation was established. Meanwhile, the laxative effect of TKP on the fecal excretion function, intestinal inflammatory response, neurotransmitter secretion and gut microbiota composition were evaluated.

**Results:**

Treatment with the TKP alleviated the constipation-associated pathological symptoms in mice through enhancing the gastrointestinal (GI) transit ratio and improving defecation function. Moreover, TKP supplementation modulated the secretion levels of neurotransmitters in the serum of mice, elevating excitatory neurotransmitters (SP, MTL and ACH), while suppressing inhibitory neurotransmitters (NO, VIP and ET-1). Additionally, TKP reduced the level of MDA, and enhanced the activities of SOD and GSH-Px, effectively attenuating oxidative stress. Under TKP administration, colonic levels of pro-inflammatory cytokines (IL-6, IL-1β and TNF-α) were significantly suppressed, while upregulating the expression of tight junction proteins (ZO-1 and occludin). Crucially, TKP increased the production of short-chain fatty acids (SCFAs), and modulated the gut microbiota by restoring its abundance and diversity in constipated mice.

**Conclusion:**

Collectively, these findings demonstrated that TKP could ameliorate diphenoxylate-induced functional constipation in mice, providing a pharmacological foundation for its development as a novel therapeutic agent against constipation.

## 1 Introduction

Functional constipation, as a kind of gastrointestinal dysfunction without organic disease, is common across all age groups. The clinical symptoms of functional constipation mainly include difficulty defecating, reduced frequency of defecation and dry stool (Vriesman et al., 2020). Studies have shown that the global prevalence of functional constipation is from 10.1% to 15.3%, and its occurrence is closely related to multiple factors such as dietary habit, mental state, psychological status, genetics and living environment (Barberio et al., 2021; Camilleri et al., 2017). Chronic constipation significantly impairs quality of life and may predispose individuals to various disorders, including cardiovascular, gastrointestinal, and neurological diseases (Bharucha and Lacy, 2020; Dong et al., 2023; Staller et al., 2022; Liu et al., 2017). Current constipation management relies on laxatives and prokinetic agents, while chronic administration may induce drug dependence (Li et al., 2024). Therefore, it is of great significance to develop safe and effective natural medicines to prevent and relieve constipation.

Polysaccharides, as a class of natural macromolecular compounds with a variety of biological activities, are widely found in plants, animals and microorganisms (Ye et al., 2023). In addition to immunomodulation, antioxidant, anti-inflammatory and other pharmacological effects (Yu Y. et al., 2018; Lu et al., 2020), polysaccharides also play an important role in regulating gut microbiota, enhancing intestinal peristalsis and improving intestinal barrier function (Zhang et al., 2021), which can effectively improve the symptoms of constipation (Jiang et al., 2020). For example, *Dendrobium officinale* polysaccharide was proved to exert laxative effect through enhancing the gastrointestinal transit ratio, regulating gastrointestinal hormone levels and increasing fecal water content in constipated mice (Luo et al., 2017). *Gastrodia elata* Blume polysaccharide significantly increased the abundance of probiotics (such as *Bifidobacterium*, *Collinsella*, *Prevotella*, and *Faecalibacterium*) and inhibited the growth of pathogenics (such as *Shigella*, *Dorea*, *Desulfovibrio*, and *Blautia*) to regulate the gut microbiota. Meanwhile, *Gastrodia elata* Blume polysaccharide was able to maintain gut health by regulating gut microbiota and increasing the production of beneficial metabolite such as short-chain fatty acids (SCFAs) (Gan et al., 2024). *Atractylodes macrocephala* polysaccharide could reduce constipation-related symptoms by restoring the balance of gut microbiota and affecting related metabolic pathways to increase the production of SCFAs and up-regulate the expression levels of 5-hydroxytryptamine (5-HT) and chromogranin A (CgA) in constipated mice (Yang H. et al., 2023). As an essential traditional Chinese medicine, Trichosanthis Fructus is the dry ripe fruits of *Trichosanthes kirilowii* Maxim or *Trichosanthes rosthornii* Harms of the Cucurbitaceae family. It is traditionally employed for its therapeutic effects in clearing heat and resolving phlegm, moistening the intestines, and promoting bowel movements (Yu X. et al., 2018). Among its main active components, *Trichosanthes kirilowii* polysaccharide (TKP) has garnered growing attention due to their pharmacological potential. Recent studies have demonstrated that TKP exhibited the diverse biological activities, including antioxidant, anti-inflammatory, immunomodulatory, lipid-lowering, and gut microbiota-regulating effects (Jiang Y. et al., 2024; Zhang et al., 2022a; Hu et al., 2020; Yang H. et al., 2023; Yang S. Y. et al., 2023). However, the preventive effect of TKP on functional constipation and its mechanism are still unclear.

Consequently, the preventive effect of TKP on functional constipation in mice was investigated. In this study, the model of functional constipation in mice induced by diphenoxylate was established. To evaluate the laxative effect of TKP, the study examined the fecal excretion function, intestinal inflammatory response, neurotransmitter secretion and gut microbiota composition. These findings provide a theoretical basis for the prevention of functional constipation by TKP.

## 2 Materials and methods

### 2.1 Materials

The flesh of *Trichosanthes kirilowii* was collected from the Pingyin County, Shandong Province. Diphenoxylate was purchased from Beijing Lihua Cun Trade Co. Ltd. Bisacodyl was obtained from Sigma-Aldrich.

### 2.2 Preparation of TKP

The crude polysaccharide from *Trichosanthes kirilowii* (TKP) was isolated using the water extraction and alcohol precipitation method, based on the previous study (Wang et al., 2024). Briefly, the flesh of *Trichosanthes kirilowii* was subjected to triple extraction with boiling water. The combined aqueous extracts were filtered to remove insoluble residues and then was concentrated. The concentrate was mixed with absolute ethanol to achieve a final ethanol concentration of 80% (v/v) for polysaccharide precipitation and stored at 4 °C overnight. Subsequently, the resulting precipitate was redissolved in distilled water, and further purified to obtain the crude polysaccharide fraction. Then, the crude polysaccharide was defatted with petroleum ether. Finally, the product was freeze-dried to obtain TKP. The yield of TKP was 0.68%. The chemical composition of TKP was total sugar (93.24%, w/w), ash (1.28%, w/w), and protein (3.87%, w/w). The monosaccharide composition of TKP was arabinose (35.32%, w/w), glucose (24.63%, w/w), galactose (22.90%, w/w), rhamnose (4.31%, w/w), mannose (6.39%, w/w), fructose (0.93%, w/w), and galacturonic acid (5.51%, w/w) (Supplementary Table 1). The content of total sugar was determined by the phenol-sulfuric acid method. The contents of ash and protein were measured using the previous methods (Sousa et al., 2022; Kielkopf et al., 2020). The monosaccharide composition was analyzed using a Thermo ICS 5000 ion chromatography system (ICS 5000, Thermo Fisher Scientific, United States).

### 2.3 Animals and experimental design

Kunming mice (female, 4 weeks old; Beijing Vital River Laboratory Animal Technology Co., Ltd.) were housed under controlled conditions (23–25 °C, 40%–60% humidity, and 12 h light/dark). All mice were acclimated for 7 days before constipation experiments and then randomly divided into six groups (*n* = 8/group) as follows: the normal control (NC) group, the model control (MC) group, the bisacodyl positive control (BC) group, the low-dose TKP (TKP-L) group, the middle-dose TKP (TKP-M) group, the high-dose TKP (TKP-H) group. Functional constipation was induced following the established method of Li et al. (2021). During the initial 14 days, the normal control (NC) and model control (MC) groups received equal volume of saline, while the bisacodyl control (BC) group was treated with bisacodyl (0.1 g/kg⋅bw/d). The experimental groups (TKP-L, TKP-M, and TKP-H) received TKP at doses of 300, 600, and 900 mg/kg⋅bw/d, respectively, based on the previous study (Chen et al., 2016). Mice were administered test compounds via oral gavage at a volume of 10 mL/kg⋅bw/d. Subsequently, functional constipation was induced in MC, BC and TKP-treated groups by oral administration of diphenoxylate (30 mg/kg⋅bw/d) for three consecutive days, based on the previous study (Li et al., 2024). The model of constipation was performed after 14 consecutive days of TKP or control administration. The NC group continued to receive an equivalent volume of saline. Body weight, food intake, and water consumption were monitored daily in all mice.

At the end of these experiments, mice were euthanized under anesthesia for the collection of blood, cecum and colon. Blood samples were centrifuged (3,000 rpm, 10 min, 4 °C) to isolate serum. Cecal contents and the proximal colon were quickly taken and stored at −80 °C, and the distal colon tissues were immersed in formaldehyde solution.

### 2.4 Cell culture and treatment

RAW264.7 macrophage cells were cultured in dulbecco’s modified eagle medium (DMEM) supplemented with 10% (v/v) fetal bovine serum (FBS) in an incubator at 37 °C with 5% CO_2_ (Arunrat et al., 2025). For toxicity test, RAW264.7 cells (2 × 10^4^ cells/well) were inoculated into 96-well plates, and then treated with different concentrations (0.025, 0.05, 0.1, 0.2, 0.4, 0.8, 1.0, and 2.0 mg/mL) of TKP for 24 h, based on the previous study (Li et al., 2025). For further study, RAW264.7 cells (5 × 10^5^ cells/well) were seeded on a six-well plate. A total of 1 μg/mL of lipopolysaccharide (LPS) was administered in model group, then TKP was added at the optimal concentration for 24 h.

### 2.5 Black fecal parameters

The determination of black fecal parameters was conducted according to the method described by Liu et al. (2019), with minor modifications. After fasting for approximately 18 h, four mice in each group randomly selected to test defecation function were fed with 10% activated carbon solution (10 mL/kg⋅bw). The mice were immediately separated into a single cage and fed with basal diet and water. Observation was continued for 6 h, and first defecation time of black feces in each mouse was recorded. The wet weight and number of black feces in mice were measured.

### 2.6 Determination of the gastrointestinal (GI) transit ratio

Gastrointestinal (GI) transit ratio was determined according to the previous method (Su et al., 2019). Briefly, all mice were administered an activated carbon suspension (10 mL/kg⋅bw). A total of 25 min’ post-administration, four mice per group were randomly euthanized by cervical dislocation following blood collection. The entire small intestine was carefully excised from pylorus to cecum and laid straight without stretching on a chilled surface. Gastrointestinal (GI) transit ratio was calculated using the formula: GI transit ratio (%) = movement distance of activated carbon/the total length of small intestine × 100.

### 2.7 Measurement of biochemical indicators

The levels of substance P (SP), motilin (MTL), vasoactive intestinal peptide (VIP), acetylcholine (ACH), and endothelin-1 (ET-1) in the serum of mice were measured by ELISA kits according to the instructions (Shanghai Enzyme linked Biotechnology Co., Ltd., China). The contents of nitric oxide (NO), superoxide dismutase (SOD), glutathione peroxidase (GSH-Px), and malondialdehyde (MDA) were analyzed using commercial ELISA kits (Beyotime Biotechnology Co., Ltd.). Inflammation-associated factors including tumor necrosis factor-α (TNF-α), interleukin-6 (IL-6), and interleukin-1β (IL-1β) in the colon of mice were measured using the corresponding kits (NeoBioscience technology Co. Ltd., China).

### 2.8 Histological and immunohistochemical analysis

Distal colon tissues were excised, rinsed with normal saline, and fixed in 4% paraformaldehyde for 24 h at room temperature. After routine dehydration, clearing, and paraffin embedding, tissues were sectioned at 4 μm, deparaffinized, rehydrated, and stained with hematoxylin and eosin (H&E) following standard procedures. Histopathological changes were observed under a light microscope (Nikon, Japan).

The expression levels of 5-hydroxytryptamine receptor 4 (HTR4) and vasoactive intestinal peptide receptor 1 (VIPR1) in colon tissues were assessed by immunohistochemistry. Briefly, the deparaffinized slides were blocked with 10% normal goat serum for 1 h. Sections were incubated with primary antibodies [anti-VIPR1 (BIOSS, 1: 300) and anti-HTR4 (Proteintech, 1: 300)], followed by secondary antibody (1: 200). Then, 3,30-diaminobenzidine (DAB) staining was visualized using a BX60 fluorescence microscope.

### 2.9 Western blotting analysis

The detailed procedure of Western blotting was conducted by the previously described (Li et al., 2021). Briefly, 20 mg of colonic tissue was homogenized in 200 μL of RIPA lysis buffer supplemented with protease inhibitor cocktail. Total protein concentration was quantified using BCA protein assay kit (Beyotime Biotechnology, China) according to the manufacturer’s protocol. Protein samples (20 μg) were resolved by 10% SDS-PAGE and subsequently transferred onto PVDF membranes. Then, membranes were blocked with 5% non-fat milk in TBST for 1 h at room temperature, then incubated overnight at 4 °C with the following primary antibodies (diluted 1: 1000): anti-ZO-1, anti-occludin, and anti-GAPDH (as loading control). After extensive washing, membranes were probed with HRP-conjugated secondary antibodies (1: 5,000 dilution) for 1 h at room temperature. Protein bands were visualized using enhanced chemiluminescence (ECL) reagent and quantified by densitometric analysis with ImageJ software (NIH, USA). The levels of relative protein expression were normalized to GAPDH.

### 2.10 Gut microbiota and short chain fatty acids (SCFAs) analysis

Total genomic DNA was aseptically extracted from cecal contents using the TIANamp Stool DNA Kit (Tiangen, Beijing, China). The V3–V4 hypervariable region of the 16S rRNA gene was amplified via PCR using universal primers (338F/806R). High-quality sequences were clustered into operational taxonomic units (OTUs) at 97% similarity using UCLUST (v1.2.22). Taxonomic classification was performed against the SILVA119 database (90% confidence threshold) in QIIME, followed by beta-diversity assessment through principal coordinate analysis (PCoA). Functional gene prediction was conducted using PICRUSt based on KEGG pathways. In addition, fecal samples (0.2 g) were subjected to gas chromatography-mass spectrometer (GC-MS) to quantify the concentrations of SCFAs.

### 2.11 Statistical analysis

All data were expressed as mean ± standard deviation (SD). Statistical analyzes and bar plots generation were performed using GraphPad Prism 8.0 (GraphPad Software, San Diego, United States). One-way ANOVA analysis followed by Tukey’s test were conducted to determine the differences between multiple groups.

## 3 Results

### 3.1 Effects of TKP on the fecal parameters in constipated mice

**FIGURE 1:**
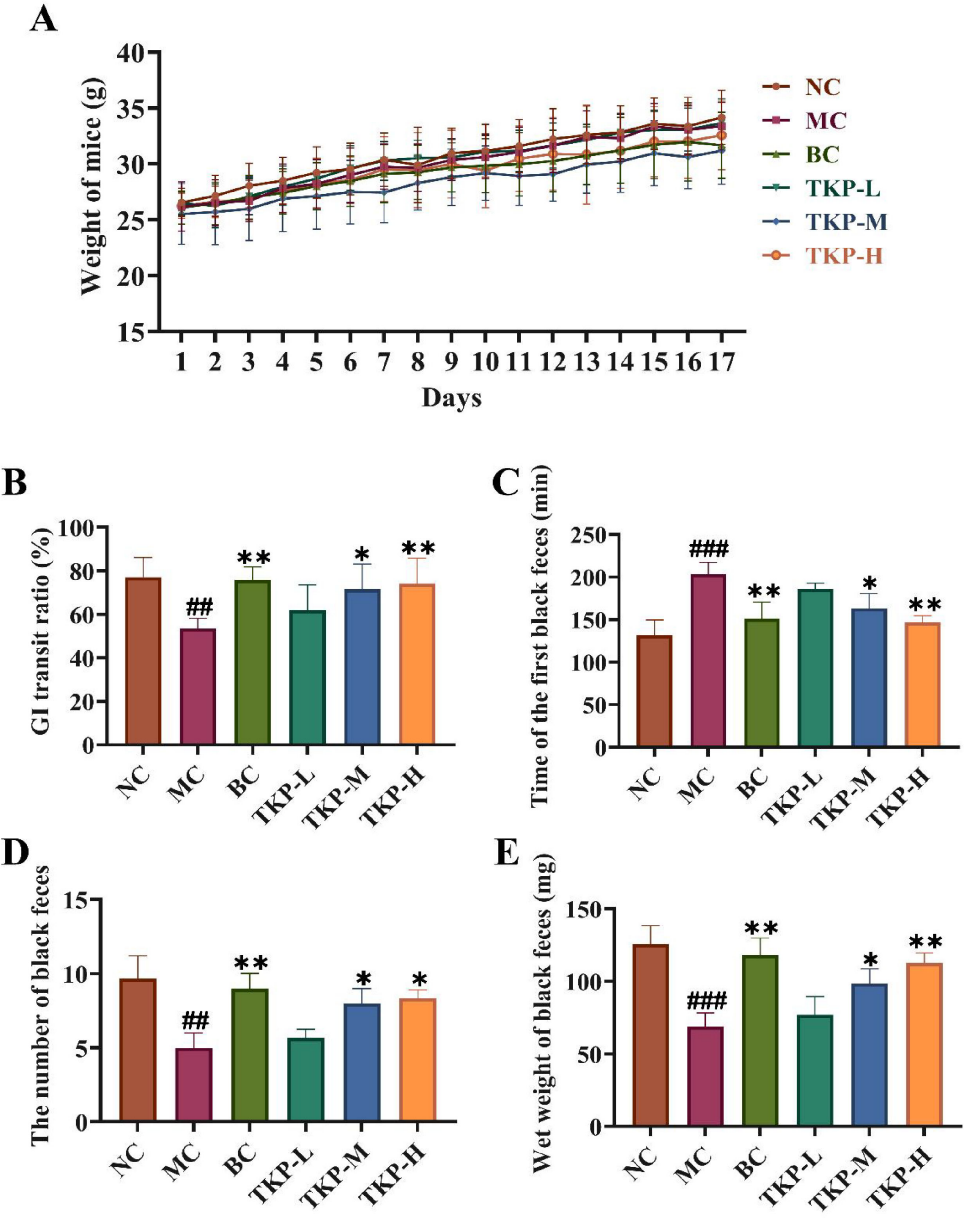
Effect of *Trichosanthes kirilowii* polysaccharide (TKP) on gastrointestinal transit function in mice with functional constipation. **(A)** Changes in body weight of mice during the experiment; **(B)** Gastrointestinal (GI) transit ratio; **(C)** The time of the first black feces; **(D)** The number of black feces; **(E)** The wet weight of black feces. NC group, normal control group; MC group, model control group; BC group, bisacodyl treatment group; TKP-L group, the low-dose TKP group (300 mg/kg⋅bw/d); TKP-M group, the middle-dose TKP group (600 mg/kg⋅bw/d); TKP-H group, the high-dose TKP group (900 mg/kg⋅bw/d). ^##^*P* < 0.01, ^###^*P* < 0.005, vs. NC group; *P < 0.05, **P < 0.01, vs. MC group.

Changes in the body weight of mice were shown in Figure 1A. Throughout the experimental period, all groups exhibited progressive body weight gain, with comparable growth trajectories consistent with normal mice development. It was shown that TKP had no significant effect on the body weight of mice in the prevention of functional constipation.

The GI transit ratio of mice in each group was shown in Figure 1B. Compared with the NC group, the GI transit ratio in the MC group mice was significantly decreased by 30.55% (*P* < 0.01), indicating that the mouse model of functional constipation was successfully established. In comparison with MC group, the GI transit ratio of mice in the TKP-L, TKP-M, and TKP-H groups was increased by 8.1%, 18.1% (*P* < 0.05), and 20.6% (*P* < 0.01), respectively. The results showed that TKP effectively ameliorated the GI transit ratio in constipated mice. As illustrated in Figures 1C–E, the time of the first black feces, the total number and wet weight of black feces within 6 h were recorded. With regard to the NC group, the time of first black feces excretion in the MC group was prolonged by 54.8% (*P* < 0.005), while the number and wet weight of black feces were reduced by 48.3% (*P* < 0.01) and 45.1% (*P* < 0.005), respectively. It indicated that the mice in the MC group experienced apparent constipation symptoms. Intriguingly, the constipation symptoms of the TKP-treated groups showed varying degrees of improvement compared with the MC group. In terms of the time of first black feces, the three different dosage treatment groups exhibited the shorter first black stool time in comparison with the MC group (*P* < 0.01), with the TKP-H group showing the most pronounced reduction (by 27.9%). This effect was comparable to that observed in the BC group. Compared with the MC group, TKP-L, TKP-M, and TKP-H administration significantly increased by 13.3%, 60.0% (*P* < 0.05), and 66.7% (*P* < 0.05) in the total number of black feces, while the wet weight of black feces elevated by 11.7%, 42.9% (*P* < 0.05), and 63.7% (*P* < 0.01), respectively. These results demonstrated that TKP could improve the defecation function of constipated mice to relieve constipation symptoms, and the effect was most obvious in the TKP-H group.

### 3.2 Effect of TKP on biochemical indicators in constipated mice and RAW264.7 cells

**FIGURE 2:**
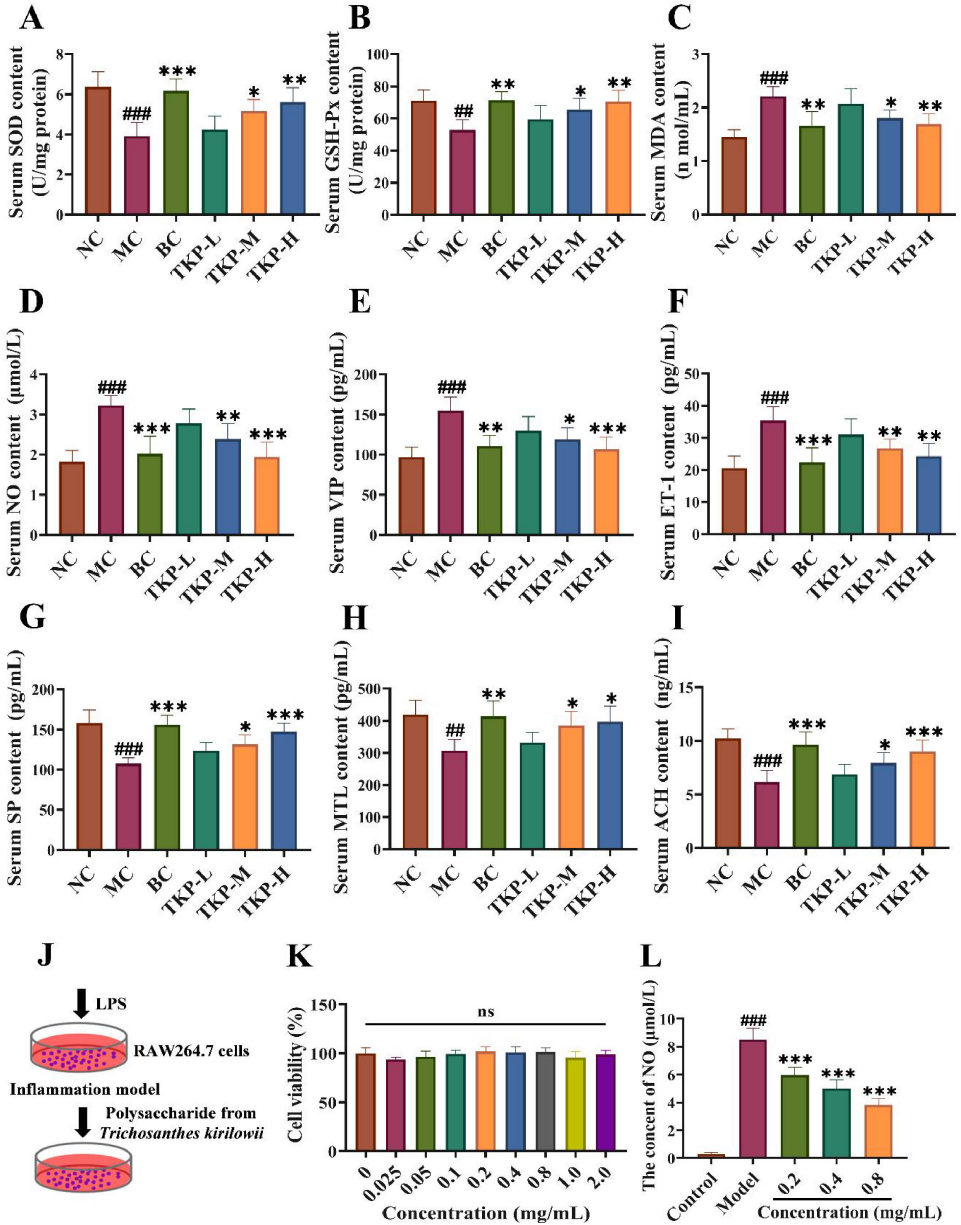
Effect of *Trichosanthes kirilowii* polysaccharide (TKP) on the levels of gastrointestinal regulation-related factors and antioxidant activity in the serum of constipated mice. **(A)** Superoxide dismutase (SOD); **(B)** Glutathione peroxidase (GSH-Px); **(C)** Malondialdehyde MDA; **(D)** Nitric oxide (NO); **(E)** Vasoactive intestinal peptide (VIP); **(F)** Substance P (SP); **(G)** Motilin (MTL); **(H)** Endothelin-1 (ET-1); **(I)** acetylcholine (ACH); **(J)** Schematic diagram of the experimental design using lipopolysaccharide (LPS)-induced inflammation in RAW264.7 cells; **(K)** Toxicity test; **(L)** The content of NO. NC group, normal control group; MC group, model control group; BC group, bisacodyl treatment group; TKP-L group, the low-dose TKP group (300 mg/kg⋅bw/d); TKP-M group, the middle-dose TKP group (600 mg/kg⋅bw/d); TKP-H group, the high-dose TKP group (900 mg/kg⋅bw/d). ^##^*P* < 0.01, ^###^*P* < 0.005, vs. NC group; *P < 0.05, **P < 0.01, ***P < 0.005, vs. MC group. ns indicates that other dosage of TKP was no significant difference vs 0 mg/mL.

The serum levels of antioxidant enzymes and neurotransmitters in constipated mice were measured. As depicted in Figures 2A–C, the contents of SOD and GSH-Px were significantly decreased as well as the increased level of MDA (*P* < 0.01 or *P* < 0.005). These findings suggested that constipation may impair intestinal antioxidant defenses, potentially inducing oxidative stress in the gastrointestinal tract. Compared with the MC group, the enzyme activities of SOD and GSH-Px were significantly increased in a dose-dependent manner in the TKP-treated groups, with the greatest increase of 53.6% and 33.4% in the TKP-H group, while the MDA content was reduced to normal value (all *P* < 0.01). It suggested that TKP could improve constipation by modulating serum-related oxidative mediators in constipated mice.

In order to explore the efficacy of TKP on the biochemical indices in constipated mice, the gastrointestinal regulation-related neurotransmitters, including NO, VIP, ET-1, SP, MTL and ACH, were assayed. As indicated in Figures 2D–I, the levels of inhibitory neurotransmitters (NO, VIP, and ET-1) in the serum were significantly higher than those in the NC group by 76.6%, 59.6%, and 71.5% (all *P* < 0.005), respectively, whereas the levels of excitatory neurotransmitters (SP, MTL and ACH) were significantly lower in the MC group compared with the NC group by 32.0%, 26.7%, and 40.0% (*P* < 0.01 or *P* < 0.005), respectively. Interestingly, the TKP-treated groups dose-dependently restored these parameters to physiological levels. It was worth noting that under the high dose administration of TKP, the levels of NO, VIP, and ET-1 were notably reduced by 39.7%, 32.0%, and 46.6% (all *P* < 0.005), respectively. Moreover, the levels of SP, MTL and ACH were obviously elevated by 37.0%, 29.3%, and 46.6%, respectively (*P* < 0.05 or *P* < 0.005). Next, the biochemical indices in LPS-induced RAW264.7 macrophage cells were tested (Figure 2J). No significant difference in cell viability under different concentrations of TKP treatment was observed (Figure 2K), indicating that TKP did not have toxic effects on RAW264.7 cells. As presented in Figure 2L, the level of NO was significantly increased in the model group when compared with the control group (all *P* < 0.005). In comparison with the model group, the levels of NO under the concentrations at 0.2, 0.4, and 0.8 mg/mL of TKP treatment were obviously decreased (all *P* < 0.005).

### 3.3 Immunohistochemical image analysis of the colon in constipated mice

**FIGURE 3:**
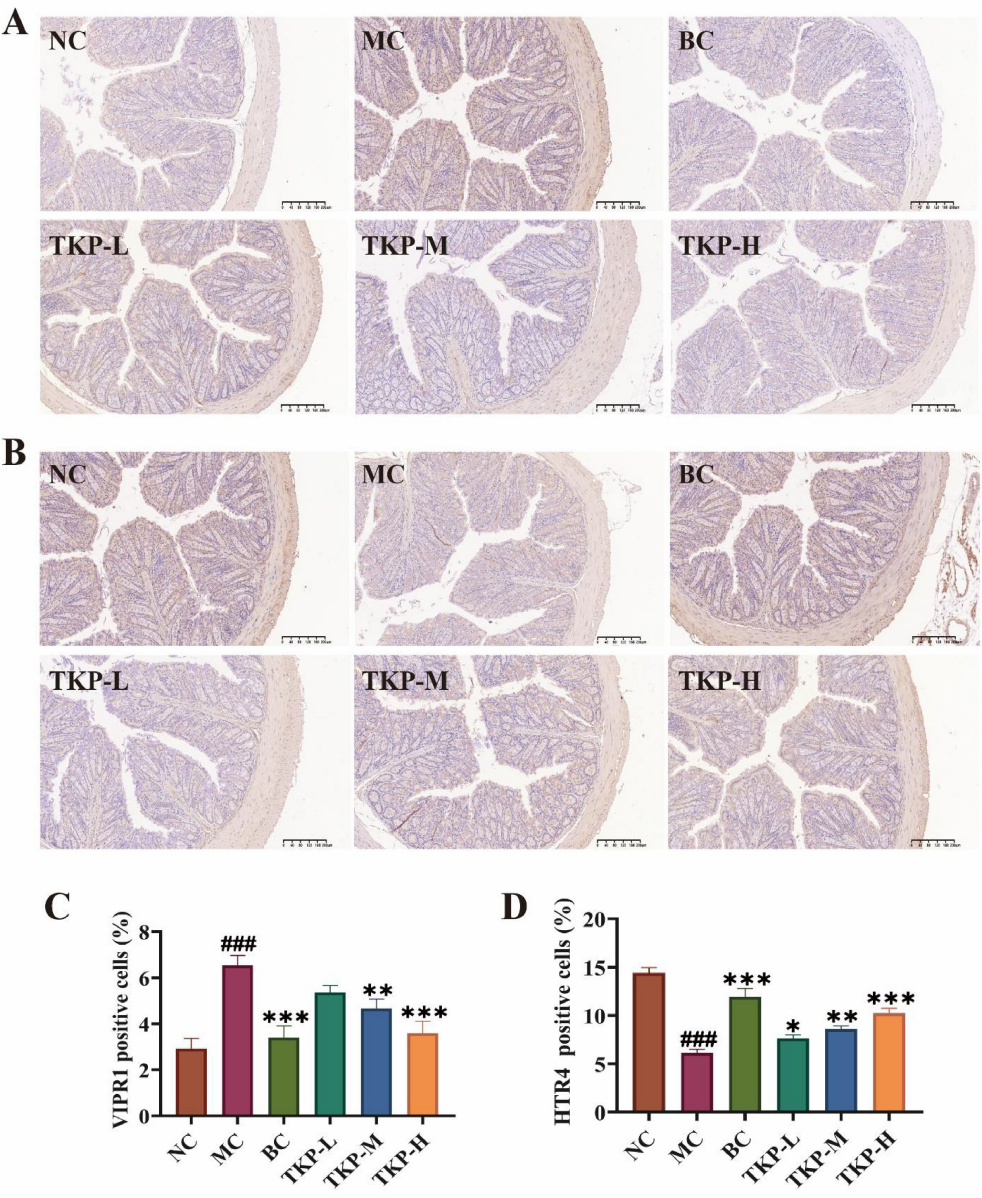
Effect of *Trichosanthes kirilowii* polysaccharide (TKP) on the expression levels of vasoactive intestinal peptide receptor 1 (VIPR1) and 5-hydroxytryptamine receptor 4 (HTR4) in the colon tissue of constipated mice. **(A)** Immunohistochemical image of VIPR1 expression; **(B)** Immunohistochemical image of HTR4 expression; **(C)** Rate of VIPR1 positive cell; **(D)** Rate of HTR4 positive cell. NC group, normal control group; MC group, model control group; BC group, bisacodyl treatment group; TKP-L group, the low-dose TKP group (300 mg/kg⋅bw/d); TKP-M group, the middle-dose TKP group (600 mg/kg⋅bw/d); TKP-H group, the high-dose TKP group (900 mg/kg⋅bw/d). ^###^*P* < 0.005, vs. NC group; *P < 0.05, **P < 0.01, ***P < 0.005, vs. MC group.

The immunostaining images analysis of VIPR1 and HTR4-positive cells in the colon tissue of constipated mice were demonstrated in Figures 3A–D. In term of VIPR1 expression, the NC group showed a low proportion of VIPR1-positive cells, and the proportion of VIPR1-positive cells in the MC group was significantly elevated by 6.5% (*P* < 0.005). Among the TKP-treated groups, the proportion of VIPR1-positive cells tended to decreased with the increase of the dose. Especially in the TKP-H group, the proportion reached about 3.5% (*P* < 0.005), close to the level of the NC group. Nevertheless, the expression level of HTR4 was opposite to that of VIPR1. After gavage of diphenoxylate, the proportion of HTR4-positive cells was lower in the MC group than that of those in the NC group. The proportion of HTR4-positive cells in the TKP-treated groups tended to increase with dose, displaying a clear dose-dependent condition. The immunohistochemical results illustrated that TKP treatment was able to up-regulate the expression level of HTR4 and down-regulate the expression of VIPR1 in the colon of constipated mice, especially at high doses.

### 3.4 Improvement of intestinal inflammatory factors and mucosal barrier by TKP in constipated mice

**FIGURE 4:**
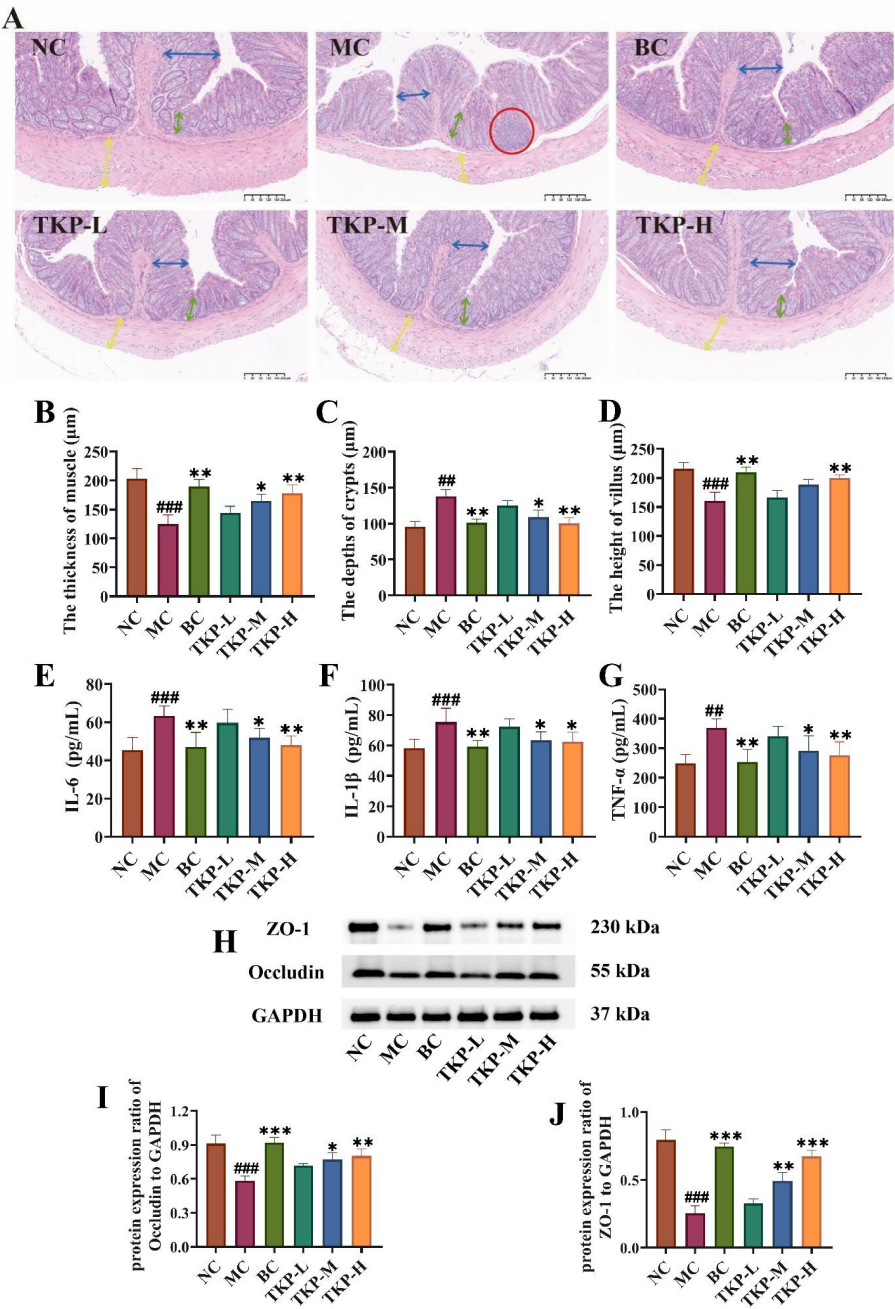
Effect of *Trichosanthes kirilowii* polysaccharide (TKP) on the levels of intestinal inflammatory factors and tight junction proteins in constipated mice. **(A)** The images of hematoxylin and eosin (H&E) staining in the colon tissues; **(B)** The thickness of muscle; **(C)** The depth of crypt; **(D)** The height of villus; **(E)** Interleukin-6 (IL-6); **(F)** Interleukin-1β (IL-1β); **(G)** Tumor necrosis factor-α (TNF-α); **(H)** Images of ZO-1 and Occludin via western blotting; **(I)** Protein expression ratio of ZO-1 to GAPDH; **(J)** Protein expression ratio of occludin to GAPDH. NC group, normal control group; MC group, model control group; BC group, bisacodyl treatment group; TKP-L group, the low-dose TKP group (300 mg/kg⋅bw/d); TKP-M group, the middle-dose TKP group (600 mg/kg⋅bw/d); TKP-H group, the high-dose TKP group (900 mg/kg⋅bw/d). ^##^*P* < 0.01, ^###^*P* < 0.005, vs. NC group; *P < 0.05, **P < 0.01, ***P < 0.005, vs. MC group.

Inflammatory response and impaired mucosal barrier in the colon tissue are important features of constipation. As shown in Figure 4A, histological examination revealed the regular mucosal folds and tightly arranged cells in the NC group. However, mice in the MC group had an abnormal colon condition, which was mainly characterized by the mucosa and submucosa exhibiting obvious signs of inflammatory infiltration. In contrast, the BC and TKP-H groups maintained intact villus architecture, exhibiting histological organization comparable to healthy controls. Morphometric analysis revealed significant reductions in the MC group mice of colonic muscular layer thickness (by 38.5%, *P* < 0.005) and villus length (by 25.7%, *P* < 0.005), along with a 43.7% increase in crypt depth (*P* < 0.01) when compared with the NC group (Figures 4B–D). Nevertheless, TKP treatment dose-dependently ameliorated these changes, especially TKP-H showing bisacodyl-equivalent activity (*P* < 0.01).

To further explore colonic inflammation in constipated mice, we measured the levels of the inflammation-related factors including IL-6, IL-1β, and TNF-α. As shown in Figures 4E–G, with regard to the NC group, the levels of TNF-α, IL-1β, and IL-6 were significantly elevated by 39.5%, 29.6%, and 47.9%, respectively (*P* < 0.01 or *P* < 0.005). After the TKP-M and TKP-H intervention, the levels of IL-6, IL-1β, and TNF-α were significantly reduced compared with the MC group (*P* < 0.05 or *P* < 0.01). ZO-1 and occludin are key tight junction proteins, playing a vital role in maintaining tight junctions between intestinal epithelial cells and safeguarding the integrity of the intestinal barrier. Thus, the expression levels of ZO-1 and occludin proteins in the colon tissue were measured (Figures 4H–J). The results indicated that the expression levels of ZO-1 and occludin were significantly lower in the MC group compared with the NC group (all *P* < 0.01). Notably, TKP treatment dose-dependently upregulated the expression levels of ZO-1 and occludin. These findings correlated with histological observation from HE-stained colon sections, indicating that TKP treatment enhanced the intestinal barrier function.

### 3.5 Effect of TKP on short chain fatty acids (SCFAs) in constipated mice

The levels of SCFAs in feces of constipated mice were measured. As summarized in Table 1, the levels of acetic acid, propionic acid, isobutyric acid, butyric acid, isovaleric acid, and valeric acid in feces of the MC group mice were significantly decreased by 37.4%, 49.2%, 61.6%, 64.1%, 78.7%, and 73.2% (all *P* < 0.01), respectively, compared with NC group. Importantly, with regard to the MC group, the contents of acetic acid, propionic acid, isobutyric acid, butyric acid, isovaleric acid, and valeric acid in the TKP-H group were significantly increased by 58.0%, 83.9%, 115.5%, 145.6%, 242.2% (*P* < 0.05 or *P* < 0.01), respectively. These results indicated that the TKP treatment gradually restored the production of SCFAs, and the TKP-H group reached a level comparable to that of the BC group.

**TABLE 1:** Effect of *Trichosanthes kirilowii* polysaccharide on short-chain fatty acids in the contents of the cecum (ng/mg).

Groups	Acetic acid	Propionic acid	Isobutyric acid	Butanoic acid	Isovaleric acid	Valeric acid
NC	320.16 ± 27.38	73.97 ± 5.44	4.04 ± 0.79	127.68 ± 22.32	2.11 ± 0.14	6.56 ± 0.95
MC	200.36 ± 23.05**	37.56 ± 11.26**	1.55 ± 0.36**	45.90 ± 17.65**	0.45 ± 0.25**	1.76 ± 0.37**
BC	305.77 ± 25.07^#^	73.09 ± 5.48^##^	3.75 ± 0.90^#^	117.84 ± 18.40^#^	1.62 ± 0.57^#^	5.85 ± 0.66^#^
TKP-L	230.12 ± 17.83	52.70 ± 11.14	2.31 ± 0.72	70.68 ± 16.17	1.13 ± 0.26	3.37 ± 1.37
TKP-M	279.25 ± 35.67	60.22 ± 7.54^#^	2.57 ± 0.49	74.60 ± 21.54	1.18 ± 0.28	3.67 ± 2.10
TKP-H	316.48 ± 54.55^##^	69.06 ± 5.65^##^	3.34 ± 0.48^#^	112.74 ± 28.56^#^	1.54 ± 0.48^#^	4.98 ± 0.72^#^

NC, the normal control group; MC, the model control group; BC, the bisacodyl positive control group; TKP-L, 300 mg/kg⋅bw/d TKP group; TKP-M, 600 mg/kg⋅bw/d TKP group; TKP-H, 900 mg/kg⋅bw/d TKP group. Data are expressed as means ± SD (*n* = 8 mice/group).

**P < 0.01 vs, NC group;

^#^*P* < 0.05 vs. MC group,

^##^*P* < 0.01 vs. MC group.

### 3.6 Gut microbiota composition analysis

**FIGURE 5:**
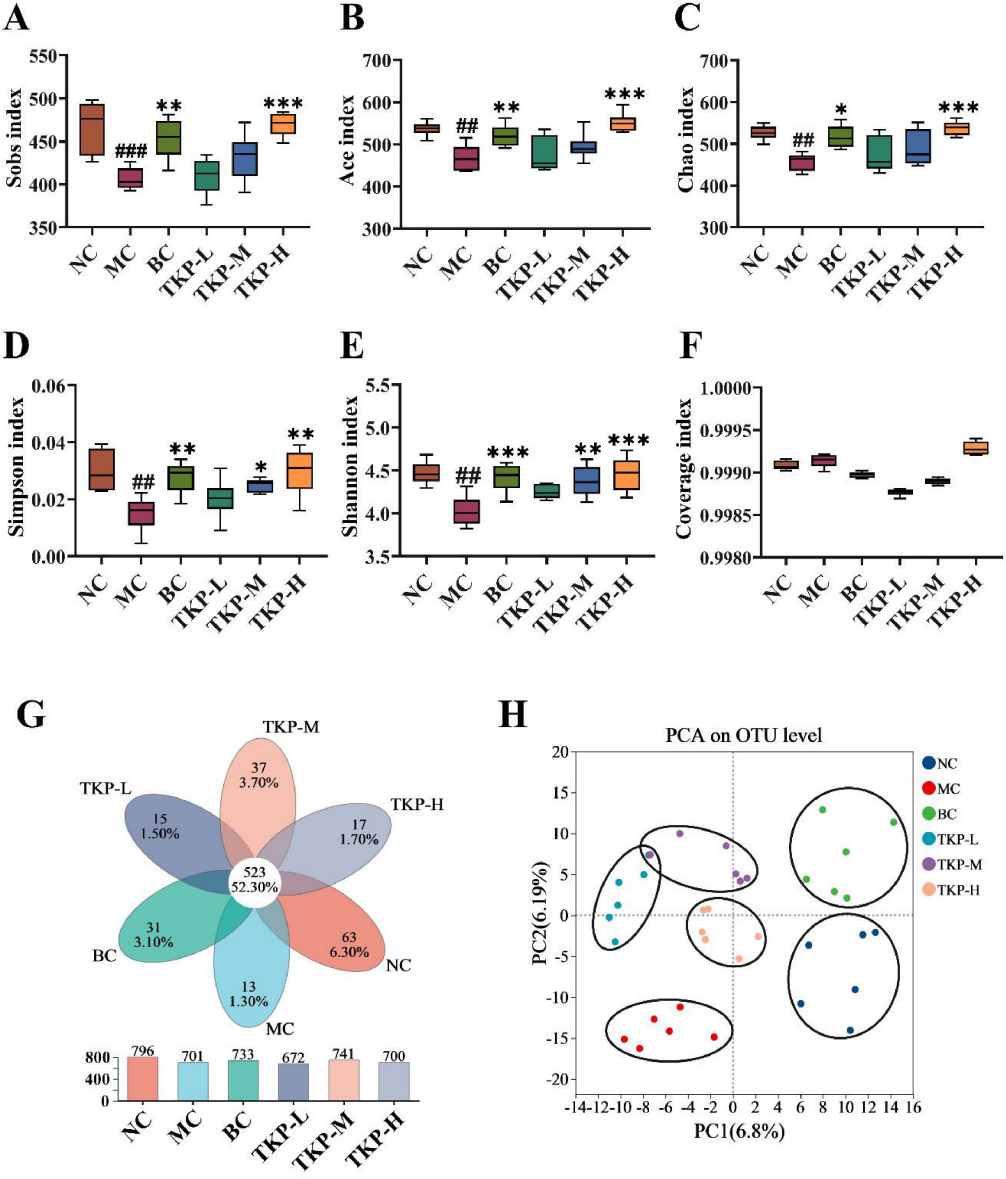
Effect of *Trichosanthes kirilowii* polysaccharide (TKP) on intestinal microbiota structure in constipated mice using 16S rDNA sequencing. **(A)** Sobs index; **(B)** Ace index; **(C)** Chao index; **(D)** Simpson index; **(E)** Shannon index; **(F)** Coverage index; **(G)** common or endemic operational taxonomic units (OTUs) to each group in Venn diagram; **(H)** Principal component analysis (PCA) analysis at OTU level. NC group, normal control group; MC group, model control group; BC group, bisacodyl treatment group; TKP-L group, the low-dose TKP group (300 mg/kg⋅bw/d); TKP-M group, the middle-dose TKP group (600 mg/kg⋅bw/d); TKP-H group, the high-dose TKP group (900 mg/kg⋅bw/d). ^##^*P* < 0.01, ^###^*P* < 0.005, vs. NC group; *P < 0.05, **P < 0.01, ***P < 0.005, vs. MC group.

To explore the influence of TKP on the gut microbiota of constipated mice, 16S rRNA sequencing was conducted in the cecal contents. Alpha diversity analysis was performed to assess species abundance and diversity. As shown in Figures 5A–E, in comparison with the NC group, Sobs, Ace, Chao, Simpson and Shannon indexes decreased in the MC group, indicating that the richness of community distribution was decreased. After the TKP treatment, Sobs, Ace, Chao, Simpson, and Shannon indexes were increased in a dose dependent manner, suggesting that TKP could restore intestinal microbiota diversity, especially the TKP-H group. The coverage index is used to assess the depth and accuracy of sequencing data. The coverage index in all groups was close to 1 (Figure 5F), indicating that this sequencing was able to realistically reflect the microbial composition of the samples. Common and unique operational taxonomic units (OTUs) between different groups were analyzed by Venn diagrams. As shown in Figure 5G, the number of common OTUs in the six groups was 523. Meanwhlie, the NC group had 63 unique OTUs, and the MC group had 13 unique OTUs, while the TKP-L, TKP-M, and TKP-H groups had 15, 37, and 17 unique OTUs, respectively. In addition, PCA analysis indicated a better taxonomic aggregation of microbial communities in all groups. In particular, the three dosing groups can be clearly distinguished from the MC group (Figure 5H).

**FIGURE 6:**
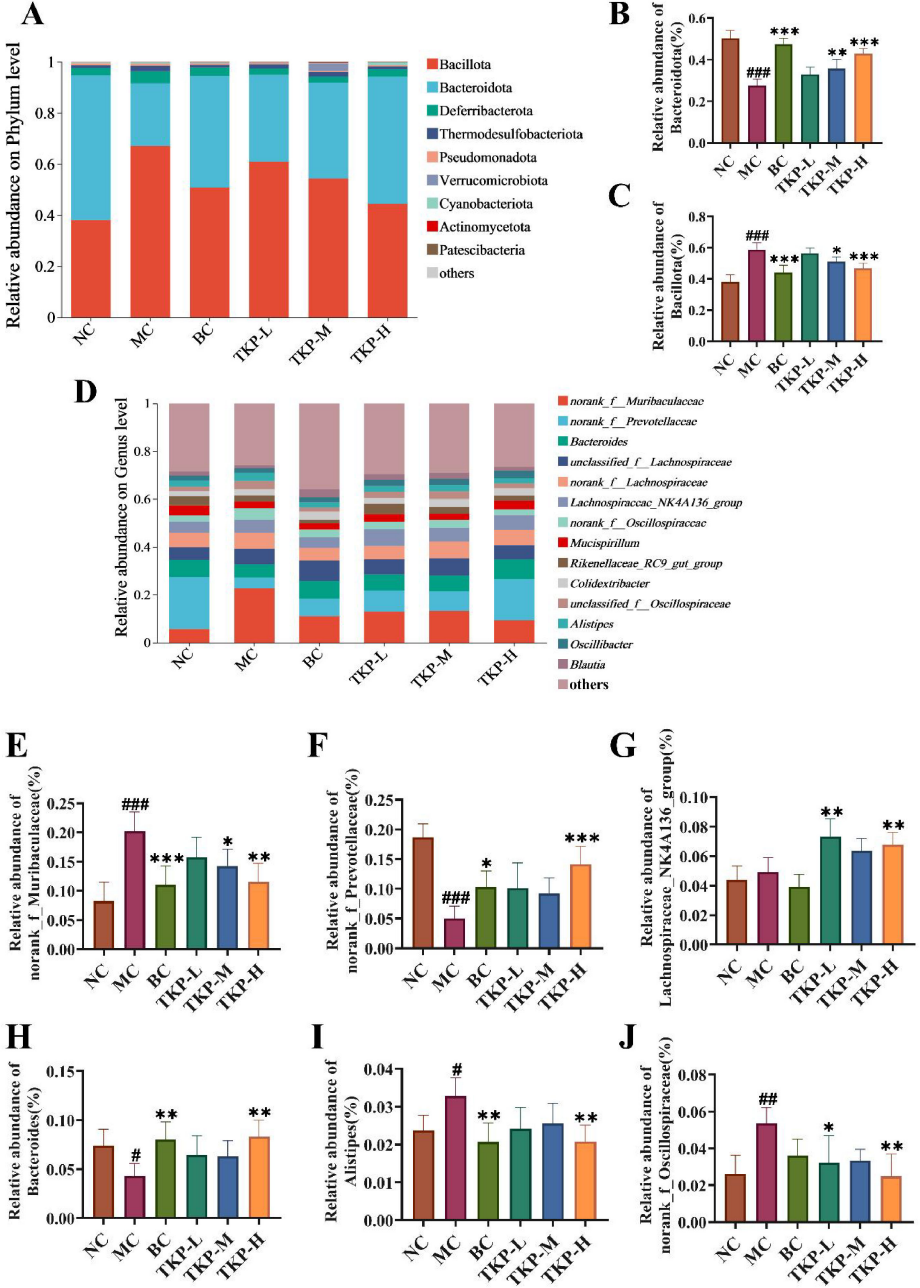
Effect of *Trichosanthes kirilowii* polysaccharide (TKP) on the relative abundance of microbial communities at the phylum and genus levels in constipated mice. **(A)** Relative abundance of gut microbiota at the phylum level; **(B)** Bacteroidota; **(C)** Bacillota; **(D)** Relative abundance of gut microbiota at the genus level; **(E)** *norank_f_Muribaculaceae*; **(F)** *norank_f_Prevotellaceae*; **(G)** *Lachnospiraceae_NK4A136_group*; **(H)** *Bacteroides*; **(I)** *Alistipes*; **(J)** *norank_f_Oscillospiraceae*. NC group, normal control group; MC group, model control group; BC group, bisacodyl treatment group; TKP-L group, the low-dose TKP group (300 mg/kg⋅bw/d); TKP-M group, the middle-dose TKP group (600 mg/kg⋅bw/d); TKP-H group, the high-dose TKP group (900 mg/kg⋅bw/d). ^#^*P* < 0.05, ^##^*P* < 0.01, ^###^*P* < 0.005, vs. NC group; *P < 0.05, **P < 0.01, ***P < 0.005, vs. MC group.

Based on the results of 16S rRNA gene sequencing in mice, the gut microbiota structural differences among the groups were further analyzed at the phylum and genus levels. As illustrated in Figure 6A, at the phylum level, Bacteroidota and Bacillota were the dominant phyla across all groups. Compared with the NC group, the MC group mice exhibited a significantly lower relative abundance of Bacteroidota (*P* < 0.005). Conversely, the relative abundance of Bacillota in the MC group was significantly higher than that of those in the NC group (*P* < 0.005). Nevertheless, TKP treatment effectively reversed the microbial imbalance (Figures 6B, C). Specifically, in the TKP-H group, the relative abundance of Bacteroidota was increased by 55.2% (*P* < 0.005), while that of Bacillota was decreased by 20.5% (*P* < 0.005), when compared with the MC group.

At the genus level, (Figure 6D) the primary genera that exhibited significant alterations were *norank_f_Muribaculaceae*, *norank_f_Prevotellaceae*, *Lachnospiraceae_NK4A136_group*, *Bacteroides*, *Alistipes*, and *norank_f_Oscillospiraceae* (Figures 6E–J). Compared with the NC group, the MC group mice exhibited an elevated relative abundances of *norank_f_Muribaculaceae*, *Alistipes*, and *norank_f_Oscillospiraceae*, while the relative abundances of *norank_f_Prevotellaceae* and *Bacteroides* were obviously reduced (*P* < 0.05, *P* < 0.01, or *P* < 0.005). Compared with the MC group, under TKP intervention, the relative abundances of *norank_f_Muribaculaceae*, *Alistipes*, and *norank_f_Oscillospiraceae* were decreased, while the relative abundances of *norank_f_Prevotellaceae*, *Lachnospiraceae_NK4A136_group*, and *Bacteroides* were increased, with significant elevation observed in the TKP-H group. These results demonstrated that TKP possessed the capability to modulate the gut microbiota, restoring its abundance and diversity.

### 3.7 Correlation between physiological indices and relative abundance of bacterial genera in constipated mice

**FIGURE 7:**
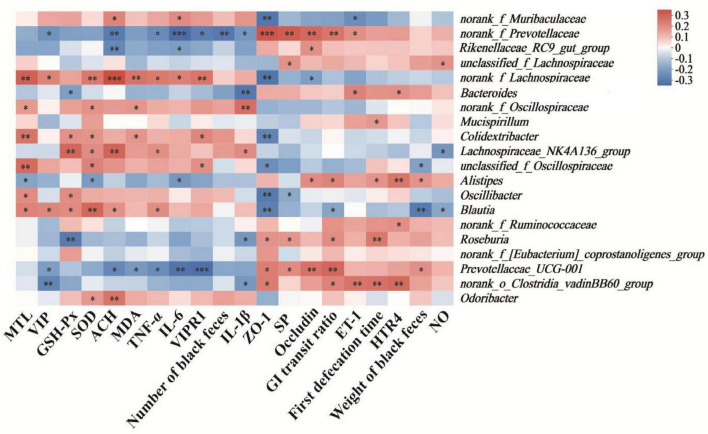
Heatmap of the correlation between physiological indices and relative abundance of bacterial genera in constipated mice. Cells marked with asterisk depict significance following spearman’s correlation, *p < 0.05, **p < 0.01, ***P < 0.005.

The Spearman correlation coefficient was calculated to investigate the correlation between physiological indices and the relative abundance of bacterial genera in constipated mice. As depicted in Figure 7, the relative abundances of *norank_f_Prevotellaceae* and *Prevotellaceae_UCG-001* exhibited the significant positive correlations with biochemical markers, including the expression levels of ZO-1 (*P* < 0.005 or *P* < 0.05) and occludin (all *P* < 0.01), the level of SP (*P* < 0.01 or *P* < 0.05), and GI transit ratio (all *P* < 0.01). In contrast, negative correlations were observed with the levels of TNF-α (all *P* < 0.05), IL-6 (*P* < 0.005 or *P* < 0.01), and the expression level of VIPR1 (*P* < 0.05 or *P* < 0.005). Moreover, the relative abundances of *norank_f_Lachnospiraceae* and *Lachnospiraceae_NK4A136_group* presented statistically positive correlations with the levels of SOD (*P* < 0.01 or *P* < 0.05) and ACH (*P* < 0.005 or *P* < 0.01). Notably, *Bacteroides* and *norank_f_Prevotellaceae* displayed inverse correlations with the level of IL-1β (*P* < 0.01 or *P* < 0.05), with *norank_f_Oscillospiraceae* and *Lachnospiraceae_NK4A136_group* showing significant positive association (*P* < 0.01 or *P* < 0.05).

## 4 Discussion

Functional constipation, a chronic condition that is now widespread, has a profound and lasting negative impact on an individual’s health. Natural polysaccharides, as a class of natural macromolecules with minimal adverse reaction, extensive bioactivity and significant therapeutic effect, have garnered considerable attention in recent years due to their notable advantages in treating functional constipation (Zhang et al., 2022b; Xu et al., 2023). Previous studies on constipation primarily focused on cases where constipation had already developed and colonic damage was present (Hu et al., 2019). Therefore, implementing preventive interventions to maintain intestinal integrity prior to gut dysfunction is physiologically imperative. Importantly, our study is the first to investigate the role of polysaccharides from *Trichosanthes kirilowii* Maxim in the prevention of constipation. We found that TKP, a major constituent of *Trichosanthes kirilowii* Maxim, can effectively ameliorate constipation symptoms by regulating gastrointestinal neurotransmitters and alleviating intestinal microbial dysbiosis, thereby satisfactorily preventing the occurrence of functional constipation.

Constipation is characterized by the difficulty in defecation and the increased defecation time (Guan et al., 2024). In our study, TKP significantly increased the number and wet weight of black feces, and GI transit ratio in constipated mice, while the time of the first black feces was shortened, thereby alleviating constipation symptoms (Figures 1B–E). The formation of intestinal oxidative stress and the destruction of the antioxidant enzyme system are important causes of constipation (Kim et al., 2021). Our study indicated that TKP-H significantly improved the serum levels of antioxidant enzymes (Figures 2A–C). Intestinal neurotransmitters play a crucial role in regulating intestinal motility. An overproduction of inhibitory neurotransmitters and an underproduction of excitatory neurotransmitters can impair intestinal motility, leading to constipation (Liu et al., 2023; Guo et al., 2023). Administration of TKP was found to enhance the levels of excitatory neurotransmitters including SP, MTL and ACH, while reducing the levels of inhibitory neurotransmitters such as NO, VIP and ET-1, as measured by serum assays in constipated mice (Figures 2D, E). VIPR1 and HTR4 play an important role in the colon (Zhang et al., 2024). The protein encoded by the VIPR1 gene is a receptor for VIP, which is engaged in the modulation of neurotransmission and endocrine function, and the HTR4 receptor has an extensive spread in the gastrointestinal tract, which is participated in the regulation of gastrointestinal peristalsis and secretion (Shi et al., 2007; Han et al., 2022). In our study, immunohistochemical analysis revealed that TKP treatment upregulated the expression of HTR4 as well as the downregulated expression of VIPR1 in constipated mice, with these modulatory effects being most pronounced at high doses (Figure 3). These findings demonstrated that TKP exerted the significant anti-constipation effects by coordinately downregulating the inhibitory neurotransmitter/receptor activity while upregulating the excitatory neurotransmission pathways.

It is the pivotal therapeutic strategies for mitigating intestinal inflammation by reducing the release of pro-inflammatory cytokine (Cai et al., 2023; Sahakian et al., 2021; He et al., 2024). Notably, the TKP-H group showed profound attenuation of colonic inflammation, evidenced by the decreased concentrations of TNF-α, IL-1β and IL-6 (Figures 4E–G). Studies have demonstrated that the pathogenesis of functional constipation is closely related to the integrity of the intestinal barrier (Jiang H. Y. et al., 2024). Intestinal barrier dysfunction increases the permeability and induces the mucosal damage, while concomitant inflammatory responses exacerbate epithelial injury, further compromising barrier integrity (Chen et al., 2020). TKP treatment enhanced the expression of the intestinal permeability-associated tight junction proteins ZO-1 and occludin in constipated mice (Figures 4H–J).

Short-chain fatty acids are substances produced primarily by anaerobic bacteria in the intestinal tract through the fermentation of undigested carbohydrates, and usually have significant physiological regulatory functions, such as protecting the intestinal mucosal barrier and inhibiting intestinal inflammation (Chen et al., 2021). Our results demonstrated that the constipated state impaired the intestinal homeostasis, leading to the reduced production of SCFAs. Crucially, TKP intervention elevated the concentrations of SCFAs, thereby creating essential biochemical conditions for intestinal integrity.

Accumulating evidence establishes the gut microbiota as a critical regulator in functional constipation pathogenesis (Yang et al., 2022). Gut microbiota dysbiosis is intimately linked to the inflammatory responses and intestinal barrier integrity. *Artemisia argyi* polysaccharide (AAP) has been demonstrated to alleviate intestinal inflammation and gut microbiota dysbiosis in mice, playing a crucial role in maintaining intestinal barrier integrity (Ning et al., 2024). To investigate the impact of TKP on the gut microbiota in constipated mice, we performed the 16S rRNA gene sequencing on cecal content of mcie. Results revealed that mice in the MC group exhibited significant microbial dysbiosis. Bacteroidota and Bacillota constitute the predominant phyla of the gut microbiota (Tsai et al., 2025). Bacteroidota rank among the primary colonizers in the gut, and their utilization of polysaccharides is essential for maintaining functional stability within the intestinal microbial ecosystem (Cheng et al., 2022). Bacteroidota can produce more polysaccharide-degrading enzymes than Bacillota, as evidenced by genomic analyzes (Lapébie et al., 2019). At the phylum level, TKP treatment altered the relative abundances of Bacteroidota and Bacillota (Figures 6B, C). At the genus level, the relative abundances of beneficial bacterial genera, including *norank_f_Prevotellaceae*, *Lachnospiraceae_NK4A136_group* and *Bacteroides* were restored, with particularly significant effects observed in the TKP-H group (Figures 6F–H). Spearman’s correlation analysis was subsequently employed to quantify relationships between physiological parameters and genus-level microbial abundances in constipated mice. Previous study has reported a positive correlation between *Bacteroides* abundance and SP level, suggesting that increased relative abundance of *Bacteroides* may contribute to ameliorating constipation symptoms in constipated mice (Wu et al., 2025). *Lachnospiraceae_NK4A136_group* exhibits potent anti-inflammatory activity and enhances mucosal restitution in the intestinal epithelium (Zhong et al., 2021). In this study, Spearman’s correlation analysis identified the significant positive correlations between *norank_f_Prevotellaceae* and *Lachnospiraceae_NK4A136_group* and the levels of ZO-1, occludin and SP, while was significantly negative correlations with the levels of TNF-α, IL-6 and VIPR1 (Figure 7). Additionally, *Bacteroides* exhibited a significant negative correlation with the level of IL-1β. These findings elucidated the mechanism for the anti-constipation effect of TKP, partially involving microbial pathway modulation.

## 5 Conclusion

In conclusion, TKP effectively facilitated the defecation function, and ameliorated the secretion levels of neurotransmitters, inflammatory response and oxidative stress in constipated mice. Importantly, TKP repaired the intestinal barrier and restored the composition of the gut microbiota. These therapeutic effects alleviated the diphenoxylate-induced functional constipation, establishing TKP as a potent dietary therapy for constipation.

## Data Availability

The original contributions presented in the study are publicly available. This data can be found here: NCBI BioProject, accession PRJNA1309862.
