# Supplementary material for: Polysaccharide from Trichosanthes kirilowii Maxim ameliorates diphenoxylate-induced functional constipation in mice
Source: Front Microbiol. 2025 Sep 23;16:1672600. doi: 10.3389/fmicb.2025.1672600 (PMC12503411; doi:10.3389/fmicb.2025.1672600)
Supplement: Supplementary file 1 [file Table_1.doc]

**SUPPLEMENTAL FILES**

**Polysaccharide from *Trichosanthes kirilowii* Maxim ameliorates diphenoxylate-induced functional constipation in mice**

Min Jiang ^1,2^, Chuangchuang Wang ^1,2^, Jian Chen ^3^ Guozhen Wu ^1,2^, Wei Liu ^2,4^, Tao Li ^2,4^*, Xiao Wang ^2,4^*

^1^ School of Pharmaceutical Sciences, Shandong University of Traditional Chinese Medicine, Jinan 250355, China

^2^ Shandong Engineering Research Center for Innovation and Application of General Technology for Separation of Natural Products, Shandong Analysis and Test Center, Qilu University of Technology (Shandong Academy of Sciences), Jinan 250014, China

^3^ Department of Traditional Chinese Medicine, Central Hospital Affiliated to Shandong First Medical University, Jinan 250014, China

^4^ Key Laboratory for Natural Active Pharmaceutical Constituents Research in Universities of Shandong Province, School of Pharmaceutical Sciences, Qilu University of Technology (Shandong Academy of Sciences), Jinan 250014, China

*Corresponding author

Xiao Wang, Shandong Analysis and Test Center, Qilu University of Technology (Shandong Academy of Science), 19 Keyuan Street, Lixia District, Jinan 250014, Shandong, China

E-mail: wangx@sdas.org; Fax: +86-531-82964889.

**SUPPLEMENTARY TABLES**

Table S1 The chemical composition of *Trichosanthes kirilowii* polysaccharide

| Relative content | *Trichosanthes kirilowii* polysaccharide |
| --- | --- |
| Rhamnose (Rha) | 4.31% |
| Arabinose (Ara) | 35.32% |
| Galactose (Gal) | 22.90% |
| Glucose (Glc) | 24.63% |
| Mannose (Man) | 6.39% |
| Fructose (Fru) | 0.93% |
| Galacturonic acid (Gal-UA) | 5.51% |
| Ash | 1.28% |
| Protein | 3.87% |
